# TRAUMA AND RESILIENCE AMONG DIRECT CARE WORKERS IN NURSING HOMES

**DOI:** 10.1093/geroni/igad104.2398

**Published:** 2023-12-21

**Authors:** Alfred Boakye, Jennifer Morgan, Antonius Skipper, Candace Kemp

**Affiliations:** University of Maryland, Baltimore, Baltimore, Maryland, United States; Georgia State University, Atlanta, Georgia, United States; Georgia State University, Atlanta, Georgia, United States; Georgia State University, Atlanta, Georgia, United States

## Abstract

Direct Care Workers (DCWs) face persistent challenges with low pay, few benefits, heavy workloads, and limited access to paid leave. Experiences associated with COVID-19 have increased the precarity of the long-term care system and have increased DCW’s vulnerability. Against the backdrop of systemic racism and political turmoil, DCWs experience high rates of health risks (e.g., burnout, anxiety disorders, depression, and post-traumatic stress disorders) leading to a reduced quality of life. Building resilience within this workforce requires understanding the protective factors, at the community, organizational, and individual levels, to manage demanding work and personal lives. Yet, existing scholarship on how to build trauma-resilient organizations and identify and cultivate individual DCW strategies to support workers remains limited. Using a socio-ecological framework, the aim of this exploratory study was two-fold: (1) examine DCWs understanding of trauma and how it affects their ability to provide care, and (2) understand the strategies and supports DCWs use to cope with trauma and what it means to be resilient. Using purposive and convenience sampling techniques, participants were recruited into the study. Semi-structured interviews with nursing assistants in nursing centers (n=25) were used to gather data and analyzed using modified grounded theory. Findings demonstrate that the impact of COVID-19, work stress, and systemic inefficiencies negatively impact care work and DCWs’ experiences. The study identified strategies that long-term care employers and government agencies can adopt to support resilience-building and ultimately empowerment practices. Implications for retention and support for staff from diverse backgrounds are also discussed.

